# Non-invasive *in vivo* monitoring of transplanted human intestinal organoids using bioluminescence

**DOI:** 10.1016/j.sopen.2026.05.002

**Published:** 2026-05-15

**Authors:** Chioma Moneme, Antonio Vinicios Alves da Silva, Prisca C. Obidike, Bryan A. Hogg, Georgia B. Brousseau, Casandra Robinson, Christian Roig-Laboy, José Kleybson de Sousa, Yuwen Zhang, Lily S. Cheng, Sean R. Moore

**Affiliations:** a Division of Pediatric Surgery, Department of Surgery, University of Virginia, Charlottesville, VA, United States of America; b Division of Gastroenterology, Hepatology and Nutrition, Cincinnati Children's Hospital Medical Center and the Department of Pediatrics of the University of Cincinnati College of Medicine, Cincinnati, OH, United States of America; c Division of Pediatric Gastroenterology, Hepatology, and Nutrition, Department of Pediatrics, University of Virginia, United States of America

**Keywords:** Graft survival, Xenotransplantation, noninvasive longitudinal *in vivo* monitoring, Human intestinal organoids (HIOs), Red-shifted luciferase, *In vivo* bioluminescent imaging, Human pluripotent stem cells (hPSCs), Mouse kidney capsule implantation, Tissue engraftment tracking

## Abstract

**Background:**

Xenotransplantation of pluripotent stem cell–derived human intestinal organoids (HIOs) into animal hosts models early human small intestinal development. However, traditional methods to assess HIO engraftment and expansion involve invasive, non-survival procedures. We developed a novel HIO platform featuring stable intestinal epithelial expression of a red-shifted luciferase, enabling sensitive, longitudinal *in vivo* tracking of HIO xenografts.

**Methods:**

Human embryonic stem cell (hESC) line H9 was lentivirus-transduced with a plasmid encoding red-shifted luciferase under a ubiquitin C promoter. After positive selection, cells were differentiated into endoderm, mid/hindgut spheroids, and HIOs. HIO maturation and luciferase expression were confirmed by immunofluorescence prior to kidney capsule implantation in 10 adult immunodeficient SCID mice. We performed serial *in vivo* bioluminescent imaging over 12-weeks, comparing radiance across time points using the Kruskal–Wallis test. Following euthanasia, HIO grafts were explanted to assess size and tissue architecture.

**Results:**

Stable transduction did not compromise the genome integrity of hESCs, pluripotency, or maturation of hESCs into HIOs. In 9 of 10 mice, HIO radiance was detectable within two weeks of implantation. Mean radiance significantly increased over time (*p* = 0.03). Bioluminescence correlated with the mass of explanted HIOs upon euthanasia (ρ = 0.923, *p* < 0.001). Serendipitously, luciferase expression was uniform in H9 hESCs but restricted to the epithelium in HIOs pre- and post-implantation.

**Conclusions:**

We successfully generated transplantable bioluminescent HIOs from pluripotent stem cells, setting the stage for dynamic monitoring of HIOs to further elucidate human intestinal development and injury response across the lifespan and other diverse applications.

## Graphical abstract

**Figure:**
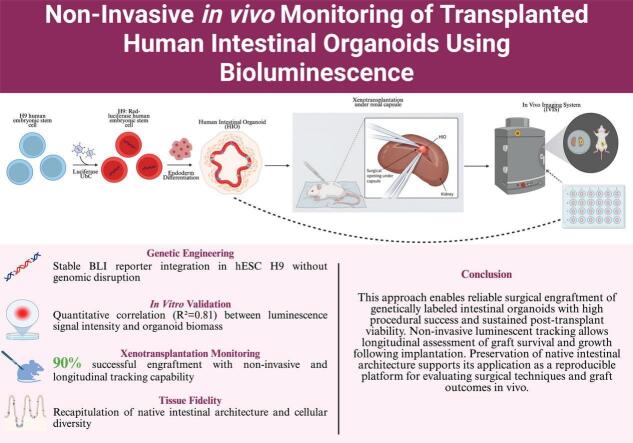

## Introduction

The gastrointestinal (GI) tract is a highly specialized organ system “highway” that integrates digestion, nutrient absorption, immune defense, barrier maintenance, and symbiotic interactions with the intestinal microbiota [1]. This functional complexity, coupled with constant exposure to dietary and microbial antigens, makes the study of intestinal homeostasis and disease pathogenesis inherently challenging. Robust experimental models capable of faithfully recapitulating the cellular architecture, physiology, and dynamic signaling of the human intestine are essential to advance a mechanistic understanding and translational discovery.

Over the past decade, advances in organoid technology have transformed GI research, enabling the generation of human intestinal organoids (HIOs) from pluripotent stem cells or primary tissue [2], [3]. HIOs derived from human pluripotent stem cells (hPSCs) self-organize into three-dimensional structures that recapitulate key epithelial and mesenchymal compartments [2], [3], [4]. HIOs exhibit a limited degree of organizational complexity when cultured *in vitro*, *e.g.*, a lack of well-defined crypt-villus architecture and features more consistent with fetal intestine. However, HIO xenotransplantation beneath the renal capsule of immunocompromised mice elicits both cellular and organizational complexity, with HIOs acquiring distinct smooth muscle layers [5] and developing a crypt-villus axis lined with a diverse epithelium containing enterocytes, goblet cells, Paneth cells, and enteroendocrine cells [4]. Functionally, these kidney capsule-matured HIOs exhibit nutrient absorption, mucus secretion, and barrier properties comparable to native intestine [3].

Beyond their utility for modeling development, HIOs have significant potential in regenerative medicine, disease modeling, drug discovery, and investigation of host-microbe interactions [8], [9], [10]. However, traditional methods for assessing the engraftment, growth and treatment response of xenotransplanted HIOs often necessitate non-survival surgeries, restricting the ability to monitor longitudinal outcomes within the same animal.

Previous strategies to address this limitation have utilized fluorescent probes to track HIOs *in vivo*, enabling real-time monitoring of engraftment without invasive procedures [11], [12], [13]. Currently, these methods rely on external light sources to excite the fluorescent reporters, which must be sufficiently intense to penetrate the surrounding tissues [14]. The light emitted by the reporters then travels back through the tissues to reach an external detector. This process may be hindered by light scattering absorption by surrounding tissues, reducing the effective penetration depth. In addition, high background noise associated with fluorescence can limit visualization capabilities.

In contrast, bioluminescence imaging (BLI) presents a non-invasive, real-time approach for tracking viable cells and tissues. BLI does not require external illumination; instead, light signals are generated through luciferase-luciferin reactions, which can be quantified with high sensitivity [15]. This allows for repeated measurements over time with minimal background interference. Due to independence of external illumination, BLI possesses enhanced tissue penetration and has been extensively utilized in cancer biology, gene therapy, and infectious disease research [16], [17]. However, the application of BLI for HIO xenotransplantation has been limited [18]. The primary technical barrier to this application is tissue attenuation, whereby bioluminescent emissions in the visible spectrum are strongly absorbed or scattered by living tissues [19], [20]. Red-shifted luciferase variants, which emit longer wavelength light with increased tissue penetration, partially overcome this limitation [15].

In this study, we engineered a human embryonic stem cell (hESC) line to stably express a red-shifted luciferase under a ubiquitin C (UbC) promoter, enabling robust and sustained epithelial-specific bioluminescent labeling following differentiation into HIOs. We used this system to longitudinally monitor HIO engraftment, growth, and compartment-specific expression after kidney capsule transplantation in immunodeficient mice. To our knowledge, this is the first demonstration of BLI-based, non-invasive tracking of transplanted HIOs *in vivo*. These findings establish a novel platform for dynamic monitoring of intestinal tissue development and survival, with broad implications for preclinical studies of intestinal regeneration and disease.

## Methods

### Establishment of stable bioluminescent reporter hESC lines

Modification of the female human embryonic stem cell (hESC) H9 line to stably express bioluminescent reporter was performed by the Pluripotent Stem Cell Facility core at CCHMC. The establishment of stable bioluminescent cell line was achieved by transduction using IVISbrite™ Lentiviral Particles (Revvity, #CLS960002), which carry the *Luciola italica* luciferase transgene under the control of the stable UbC promoter. Recipient hESC cells line H9 (WiCell, #WA09) were maintained in mTeSR1™ Complete Medium (Stemcell Technologies, #85850) supplemented with 4 μg/mL hexadimethrine bromide, using a multiplicity of infection (MOI) of 0 and 1 for 24 h.

Following transduction, multiple discrete puromycin-resistant clones were selected for further subcultivation. A single stable clone (H9:RLuc) was selected for all subsequent experiments and propagated in puromycin 2μg/mL (InvivoGen, #ant-pr-1). The pluripotency of the transduced H9:Rluc cell line was confirmed by assessing the expression of undifferentiation markers, including Nanog, Sox2, and Oct-3/4, and further validated by inducing intestinal differentiation [21].

### Karyotype analysis

A karyotype analysis of the stable clones of H9 line after transduction (H9:RLuc) was performed by Pluripotent Stem Cell Facility core at CCHMC using the classical G-banding methods as previously published. Briefly, cells were cultivated in the 6-wells-plate previously coated with hESC qualified Matrigel™ (Corning, #354277) and fed with mTeSR1™ Complete Medium until colonies reach about 80% confluence. Cells were arrested in metaphase by treatment with colcemid and disrupted with 0.075 M KCl solution. Cells were then fixed in methanol:acetic acid and dropped onto glass slides, treated with trypsin and stained with Giemsa dye, producing characteristic alternating dark and light banding patterns. The resulting G-banded spreads were analyzed under microscopy for karyotype determination and identification of chromosomal aberrations.

### Real-time bioluminescence monitoring assay

The functionality of the bioluminescent reporter system in the transduced line was confirmed by use of an incubating luminometer KronosDio AB-2550 (Atto, Japan). H9:RLuc cells were plated in 35 mm Matrigel-coated dishes at three different seeding densities, corresponding to 1-fold (high), 1:4-fold (medium), and 1:8-fold (low) dilutions of the initial cell suspension at passage. Parental H9 cells (without the reporter) were included as a negative control to assess background luminescence. All cultures were maintained in complete mTeSR-1 medium supplemented with 200 μM D-Luciferin. Bioluminescence was continuously monitored for six days, with cells cultured directly in the luminometer throughout the assay.

### Differentiation of H9:RLuc into HIOs

Differentiation of H9:RLuc cells into human intestinal organoids (HIOs) was performed following previously published methods [22]. To induce definitive endoderm differentiation, H9:RLuc cells were cultured for 24 h in RPMI 1640 supplemented with 100 ng/mL Activin A (R&D Systems, #BT-ACTAH-050) and 20 ng/mL rhWnt3a (R&D Systems, #5036-WN-010/*CF*). Cells were then cultured for an additional 48 h in RPMI 1640 containing 100 ng/mL Activin A, 8 ng/mL rhFGF2 (R&D Systems, #252057), and 0.2% defined fetal calf serum (dFCS). Definitive endoderm induction efficiency was assessed on day 3 by immunofluorescence staining for SOX17 and FOXA2 expression.

After reaching the definitive endoderm stage, cells were induced to the mid/hindgut stage by treating for four days with RPMI 1640 medium containing 500 ng/mL rhWnt3a and 500 ng/mL rhFGF4 (Sinobiological, #103663–318). This treatment led to the spontaneous formation of mid/hindgut spheroids that were collected and plated in Growth Factor Reduced Matrigel (Corning, #356231) and maintained in the Sato crypt culture medium.

The Sato crypt culture medium consisted of Advanced DMEM (Gibco, #12634028), B27 + Insulin (1×) (Gibco, #12587–10), N2 Supplement (1×) (Gibco, #17502–048), 15 mM HEPES Buffer (Gibco, #15630106), and Penicillin-Streptomycin (1×) (Gibco, #15140122). This medium was further supplemented with 50 ng/mL rhEGF (Sinobiological, #GMP-10605-HNAE), 500 ng/mL rhRSpondin1 (Sinobiological, #11083-HNAS), and 100 ng/mL rhNoggin (Sinobiological, #10267-HNAH).

### Kidney capsule transplantation

Ten adult NOD-SCID interleukin-2 receptor gamma null (NSG) mice were utilized for these experiments. The mice were procured from the Jackson Labs (strain: 005557) and consisted of 5 males and 5 females. All mice were housed in a pathogen-free animal facility at either the University of Virginia (UVA) Medical Center or Cincinnati Children's Hospital Medical Center. Handling procedures were conducted humanely in accordance with the National Institutes of Health (NIH) Guide for the Care and Use of Laboratory Animals. All experiments involving animals were approved by the Institutional Animal Care and Use Committee at both the University of Virginia (Approval number 4150) and Cincinnati Children's (Approval number IACUC2024–0112).

NSG mice were provided food and water *ad libitum* before and after surgery. A single matured HIO was transplanted under the mouse's left kidney capsule, according to previously published protocols [8]. In brief, mice were anesthetized with 2% inhaled isoflurane. The abdominal wall was prepped in a sterile fashion with 70% isopropyl alcohol. A 1–2 cm left subcostal incision was made to gain access to the retroperitoneal cavity and expose the left kidney. A small subcapsular pocket was created, and the HIO was inserted inside. The kidney was returned to its retroperitoneal space, and piperacillin/tazobactam (100 mg/kg, Eugia) was administered intraperitoneally. The skin was closed in double layers with absorbable sutures, and ketoprofen 5 mg/kg (Zoetis, New Jersey, USA) was administered intraperitoneally for pain control.

### *In vivo* bioluminescence imaging

*In vivo* bioluminescence imaging was conducted using the LagoX system (Spectral Instruments Imaging, Tucson, AZ), equipped with a back-illuminated, cooled CCD camera. Prior to imaging, mice were anesthetized with 2% inhaled isoflurane. Subsequently, 30 mg/kg D-luciferin (Revvity) was administered intraperitoneally for a working dose of 150 mg/kg. Immediately following administration, animals were then positioned in the prone position on the imaging platform and imaged every 2 min for up to 35 min to determine the mean time to peak bioluminescence. At the end of imaging, regions of interest (ROIs) were drawn and peak and time-integrated bioluminescent flux (photons/s) were quantified using Aura Imaging Software (Spectral Instruments Imaging, USA), and consistently, bioluminescent signal was found to peak 13–20 min following luciferin injection. Thus, for all animals, imaging sessions were performed within this 13–20 min post-injection time frame.

### Bioluminescent live-cell imaging

Bioluminescent live imaging of H9:RLuc cells, mid-hindgut spheroids and HIOs before implantation were performed with a Ti2 Spectra Microscope (Nikon, Japan). Before imaging, the medium of cells and HIOs was supplemented with D-luciferin for a final concentration of 200 nM.

For each sample observed under the microscope, two images were acquired. The general structure of samples was recorded with phase contrast microscopy and the emitted bioluminescence was recorded with an Andor iXon 897 EMCCD camera (Oxford Instruments) attached to the microscope using an exposure time of 5 min. The bioluminescent image was pseudocolored and then overlaid to the correspondent phase contrast image.

### Immunofluorescence staining

For immunofluorescence analysis of H9:RLuc, cells were fixed with 4% paraformaldehyde (PFA) for 30 min at room temperature, followed by three washes with phosphate-buffered saline (PBS) and then permeabilized with 0.5% Triton X-100 for 30 min at room temperature. Cells were then blocked with 0.5% Triton X-100 and 5% normal donkey serum for 45 min. Primary antibodies were applied overnight at 4 °C according to the dilutions provided in the supplementary methods, followed by three PBS washes and incubation with secondary antibodies for 1 h at room temperature. Nuclei were counterstained with DAPI, and cells were washed three additional times with PBS. Fluorescent images were acquired using a Ti2 Spectra Microscope (Nikon, Japan). Primary and secondary antibody details and dilutions are provided in the supplementary materials (Table S1).

### Immunohistochemistry

Immunohistochemistry was performed on 4 μm sections of FFPE tissue. Slides were deparaffinized in xylene and rehydrated through a graded series of ethanol. Antigen retrieval was conducted using a citrate buffer (pH 6.0) in a pressure cooker for 15 min. Endogenous peroxidase activity was blocked using H_2_O_2_, and non-specific binding sites were blocked with blocking serum before incubation with primary antibody. Sections were incubated with mouse anti-Chromogranin A (Roche Diagnostics # LK2H10) or Anti-Villin antibody [SP145] (Abcam #ab130751) primary antibodies, followed by appropriate HRP-conjugated secondary antibodies, and chromogenic detection. Slides were counterstained with hematoxylin, dehydrated and mounted for microscopic evaluation.

### *Ex vivo* tissue processing and analysis

Mice were euthanized at different timepoints to evaluate change in HIO size over time up to 12 weeks post-implantation. Following euthanasia, the left kidney containing the HIO was explanted and weighed. The mass of the HIO was estimated by subtracting the total mass of the HIO and left kidney from the mass of the left kidney alone for an age- and size-matched mouse. The HIO cross-sectional area was measured using the tracing tool in ImageJ 1× Version 1.54. [24] The explanted HIOs were then fixed in 4% paraformaldehyde (PFA), and embedded in paraffin. Tissue was sectioned at 4 μm, mounted on glass slides, deparaffinized, and rehydrated in ethanol and phosphate-buffered saline stepwise in preparation for immunohistochemical analysis, according to previously published protocols [8], [25]. For immunohistochemistry, sections were blocked using 5% donkey serum for 30 min and incubated with primary antibody overnight at 4 °C.

Next, the slides were washed and incubated in a blocking buffer containing secondary antibodies for 2 h at room temperature. Imaging was performed with a Ti2 Spectra Microscope (Nikon, Japan).

### Data and statistics analysis

Because the data were non-Gaussian, nonparametric methods were applied. Associations were assessed using Spearman's rank correlation (ρ) between: (i) bioluminescence signal intensity and cell number, (ii) cell viability and cell number, and (iii) bioluminescence signal intensity and each of the number of HIOs and explanted HIO mass. Radiance measured at multiple time points was compared using the Kruskal–Wallis test followed by Dunn's *post hoc* pairwise comparisons. The relationship between explanted HIO biomass (mg) and *in vivo* radiance was assessed using Spearman's rank correlation. To characterize the apparent exponential relationship, a log-linear regression model was fitted with radiance log_10_-transformed to satisfy linearity assumptions. The fitted model was:

$$\mathrm{lo}g_{10}\;\left(\mathrm{radiance}\right)=\beta_{0}+\beta_{1}\times \mathrm{HI}O_{-}\mathrm{biomass}+\epsilon$$

Model parameters (β₀, β₁) were estimated by ordinary least squares. Data were plotted with explanted HIO biomass on a linear x-axis and *in vivo* radiance on a log₁₀-transformed y-axis. The fitted regression line with 95% confidence band was overlaid. All analyses were performed in R (v4.5), with visualization using *ggplot2*. All tests were two-sided, with *P* ≤ 0.05 considered statistically significant.

## Results

### Stable bioluminescent hESC line maintains pluripotency and continuous signal output

**Fig. S1:**
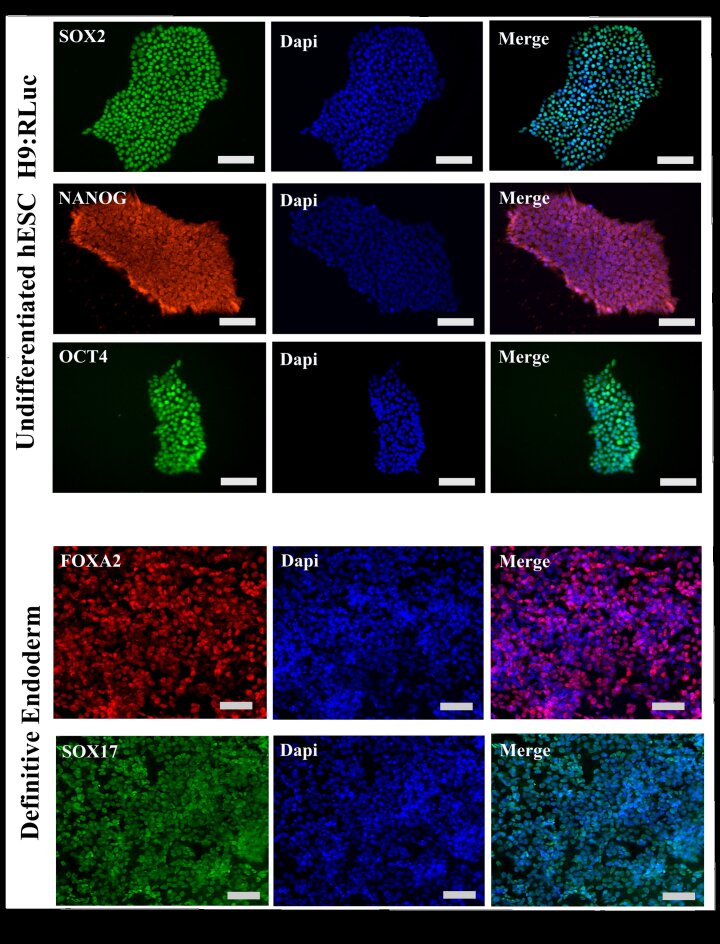
Immunofluorescence of pluripotency and Definitive endoderm (DE)-specific markers at the end of differentiation stage I. Scale bars = 100 μm.

A plasmid encoding a red-shifted luciferase reporter was stably integrated into the genome of H9 human embryonic stem cells (hESCs), generating a bioluminescent cell line (H9:RLuc). The modification did not alter karyotype, as no abnormalities were detected by G-banding (~400–800 band resolution), and did not compromise pluripotency, with cells retaining Nanog, Oct3/4, and Sox2 expression as confirmed by immunofluorescence (Supplementary Fig. 1S).

**Fig. 1:**
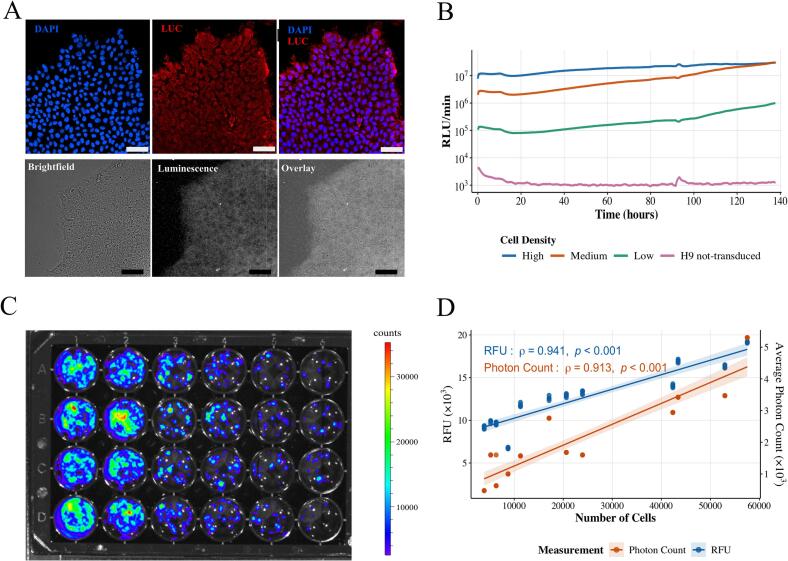
Validation of a Stable Bioluminescent Reporter in hESCs (A) Immunofluorescence staining of H9:RLuc cells confirms cytoplasmic luciferase expression (red) with DAPI nuclear counterstain (blue) (top). Bottom: Live-cell bioluminescence imaging overlaid on brightfield. Scale bars, 200 μm. (B) Real-time bioluminescence monitoring of H9:RLuc cells plated at high, medium, and low densities, showing stable signal output over time. (C) Standard curve correlating bioluminescence intensity with cell number. A representative pseudocolor image of photon counts in a 24-well plate is shown. (D) Dual-axis analysis of bioluminescent signal (orange) and cell viability (resazurin reduction; blue) as a function of cell number. (For interpretation of the references to colour in this figure legend, the reader is referred to the web version of this article.)

Undifferentiated H9:RLuc cells exhibited robust bioluminescence in the presence of D-luciferin (Fig. 1A), with luciferase expression confirmed by immunofluorescence (Fig. 1B). To evaluate long-term stability, cells were cultured in an incubator luminometer (KronosDio) with continuous luciferin supply. Bioluminescence remained stable over several days without photobleaching, and the signal was constant across the day–night cycle, suggesting insensitivity to circadian fluctuations. Bioluminescent signal intensity increased in proportion to live-cell biomass, as verified by a standard optical curve relating photon counts to cell number (Spearman ρ = 0.913, *p* < 0.001; Fig. 1C and D).

### Bioluminescence intensity correlates with human intestinal organoid biomass *in vitro*

**Fig. 2:**
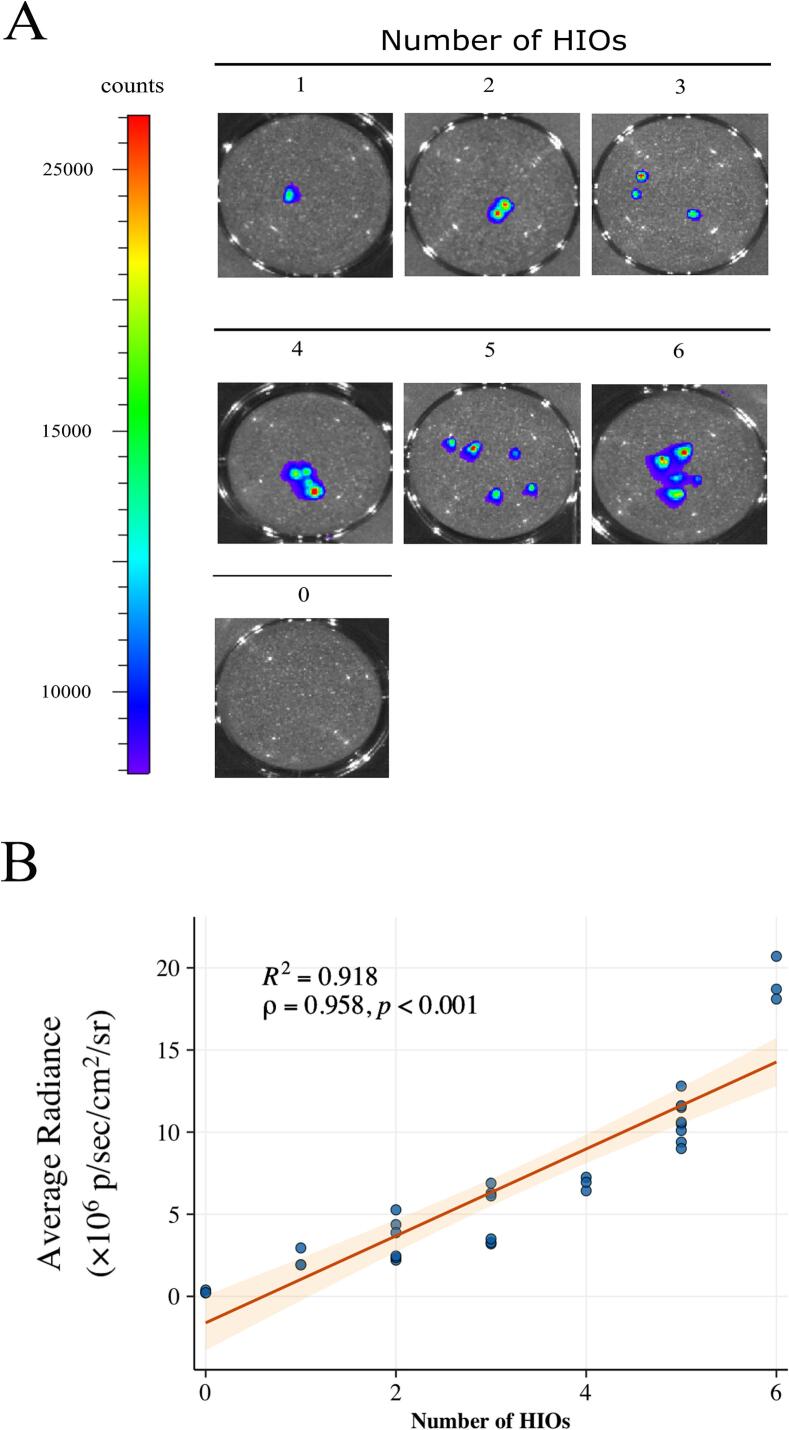
Bioluminescence intensity correlates with human intestinal organoid (HIO) biomass *in vitro*. (A) Representative composite images of mature HIOs derived from H9:RLuc cells. Brightfield images are overlaid with pseudocolored bioluminescence signal. The scale bar indicates radiant intensity. (B) Scatterplot analysis showing the correlation between total bioluminescence and the number of HIOs per well. The orange line represents a linear regression fit with a 95% confidence interval (shaded region). Spearman's rank correlation coefficient Spearman's rank correlation coefficient (ρ) and *p* value are indicated.

The reporter signal persisted through differentiation to mature human intestinal organoids (HIOs), which emitted bioluminescence upon D-luciferin exposure (Fig. 2A). Luminescence intensity scaled linearly with the number of HIOs per well (Spearman ρ = 0.96, *p* < 2.2 × 10^−16^; Fig. 2B), indicating that BLI can serve as a quantitative proxy for total HIO biomass in culture.

### Reporter expression becomes restricted to the intestinal epithelium after differentiation

**Fig. 3:**
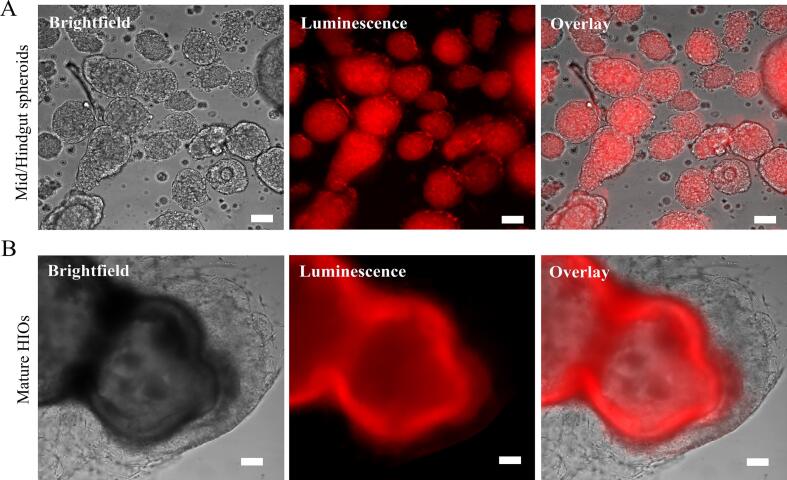
Restriction of luciferase activity to the epithelial compartment during HIO maturation. (A) Uniform bioluminescence in mid/hindgut spheroids. (B) In mature HIOs, the luciferase signal is confined to the intestinal epithelium, with no activity in the surrounding mesenchyme. Images acquired 10 min after D-luciferin addition; signal is pseudocolored red. Scale bars, 100 μm. (For interpretation of the references to colour in this figure legend, the reader is referred to the web version of this article.)

Luciferase expression was uniform in undifferentiated hESCs and persisted throughout mid- and hindgut spheroid stages (Fig. 3A). Upon terminal differentiation into HIOs, however, bioluminescence became confined to the epithelial compartment, with no detectable signal in the surrounding mesenchyme (Fig. 3B). This serendipitous compartmentalized expression was consistent across multiple independent differentiation batches and confirmed by histological analysis (Fig. 5C).

### Bioluminescence enables non-invasive tracking of HIO engraftment and growth *in vivo*

HIOs expressing intestinal epithelial markers were transplanted beneath the left renal capsule of immunodeficient mice (*n* = 10), with all animals surviving the procedure. At two weeks post-transplantation, bioluminescence was detected at the graft site in 9/10 mice, and explant analysis confirmed HIO presence in these same animals.

**Fig. 4:**
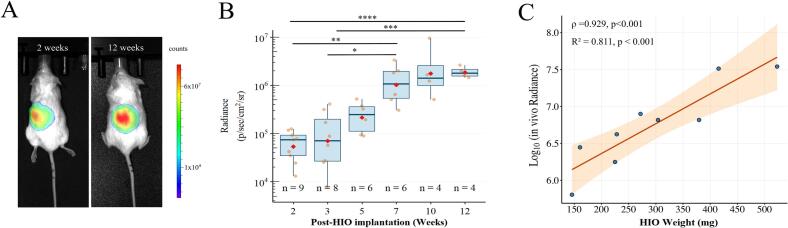
Bioluminescence Imaging Enables Longitudinal Tracking of Transplanted HIOs. (A) Representative BLI of immunodeficient mice at 2 and 12 weeks following transplantation under the renal capsule. Images were acquired 13–20 min after intraperitoneal D-luciferin injection. The colour scale indicates bioluminescence intensity. (B) Longitudinal quantification of bioluminescence signal at the graft site (detectable in 9 of 10 animals). Box-and-whisker plots display the median (red diamonds), interquartile range (box), and 1.5 × \times× IQR (whiskers), with individual data points in orange. Asterisks denote statistical significance (0.05 by Kruskal–Wallis test with Dunn's *post hoc* multiple-comparisons procedure). (C) Linear regression analysis of log_10_-transformed radiance *versus* HIO biomass (R^2^ = 0.811, *p* < 0.001). Spearman's rank correlation (ρ = 0.929, *p* < 0.001) confirms a significant positive relationship . (For interpretation of the references to colour in this figure legend, the reader is referred to the web version of this article.)

**Fig. 5:**
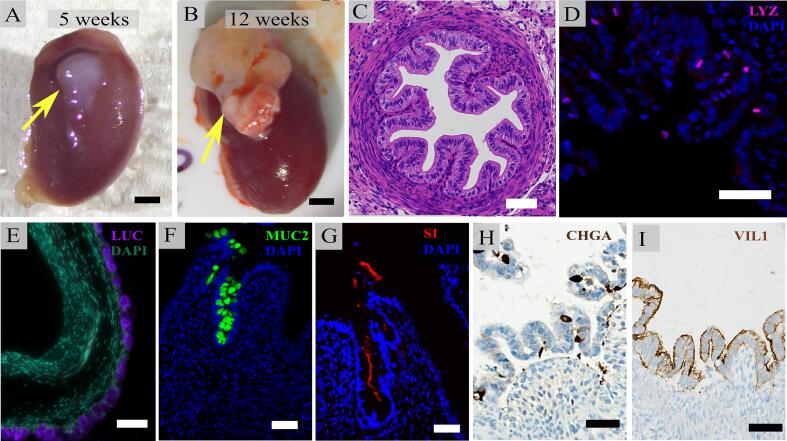
Histological characterization of explanted human intestinal organoids (HIOs). (A,B) Gross images of mouse kidneys at 5 weeks (A) and 12 weeks (B) post-transplantation; arrows indicate HIOs implanted beneath the renal capsule. Scale bar: 2 mm. (C) Hematoxylin and eosin (H&E) staining of HIOs explanted at 12 weeks. Scale bar: 50 μm. (D) Immunofluorescence staining for lysozyme indicates Paneth cell presence in explanted HIOs. Scale bar: 50 μm. (E) Immunofluorescence staining for luciferase (LUC) demonstrates epithelial-restricted reporter expression in explanted HIOs. Scale bar: 50 μm. (F) Immunofluorescence staining for Muc2 indicates goblet cell differentiation in transplanted HIOs. Scale bar: 50 μm. (G) Immunofluorescence staining for sucrase-isomaltase (SI) reveals mature enterocyte differentiation. Scale bar: 50 μm. (H) Immunohistochemistry staining for chromogranin A (CHGA) shows enteroendocrine cell differentiation. Scale bar: 50 μm. (I) Immunohistochemistry staining for villin (VIL1) demonstrates absorptive lineage presence. Scale bar: 50 μm.

Longitudinal bioluminescence imaging demonstrated a progressive increase in mean radiance (photons s^−1^ cm^−2^ sr^−1^). Because radiance values were non-normally distributed, data are summarized as median (IQR) at each time point. At week 2 (*n* = 9), median radiance was 74,300 (34,700–92,400). At week 5, the median increased to 257,665.36 (89,000-518,000). By week 12, values reached 1,800,000 (1,450,000–2,047,500) (Fig. 4A–B). Correspondingly, HIO cross-sectional area increased from 10.13 ± 3.8 mm^2^ at 5 weeks to 39.78 ± 9.5 mm^2^ at 12 weeks (*n* = 6, *p* = 0.02) (Fig. 5A, B), and mean mass increased from 0.14 ± 0.09 g to 0.26 ± 0.09 g (Fig. 4C). Bioluminescence signal intensity correlated positively with explant HIO weight, (Spearman ρ = 0.929, *p* < 0.001).

Linear regression analysis of log_10_-transformed radiance revealed a significant exponential relationship between *in vivo* bioluminescence signal intensity and HIO biomass (R^2^ = 0.811, p < 0.001). The model demonstrated high explanatory power, with biomass accounting for 81.1% of the variance in radiance. The estimated slope (β₁ = 0.00404, 95% CI: 0.00229 to 0.00578) indicates that for every 1 mg increase in HIO biomass, *in vivo* radiance increases by approximately 0.93%. Consequently, HIO biomass can be estimated from *in vivo* radiance values using the inverse function.

### Bioluminescent reporter expression does not impact HIO maturation *in vivo*

Histological analysis of explanted HIOs revealed differentiation to intestinal tissue, with gross architecture and complexity similar to previously published descriptions [5]. Maturation was maintained alongside the luciferase expression (Fig. 5E). Histological analysis of explanted HIOs *via* hematoxylin and eosin staining (H&E) revealed a well-organized architecture comprising connective tissue, layers resembling smooth muscle, and prominent villus-like structures (Fig. 5C). Immunohistochemistry and immunofluorescence showed the presence of multiple specialized, mature epithelial cell types, including absorptive lineages, such as sucrase-isomaltase^+^ enterocytes and Villin1^+^ absorptive cells (Fig. 5G, I). The presence of Lysozyme confirmed by immunofluorescence suggests presence of secretory lineages, comprising Paneth cells (Fig. 5D); enteroendocrine cells, which expressed Chromogranin A (Fig. 5H), and Muc2^+^ goblet cells (Fig. 5F). These findings confirm transplanted HIOs can survive, grow, and mature *in vivo* while maintaining bioluminescence.

## Discussion

This study demonstrates, for the first time, that bioluminescence imaging (BLI) can be used as a non-invasive, longitudinal method to evaluate engraftment, survival, and growth of transplanted human intestinal organoids (HIOs) *in vivo*. We show that BLI can confirm successful engraftment as early as two weeks post-transplantation and track continued HIO growth for up to twelve weeks. The sustained, stable light emission observed both *in vitro* and *in vivo* highlights a major advantage of bioluminescent over fluorescent reporter systems, which are prone to photobleaching and decreased expression stability under constant excitation [26].

Engraftment of intestinal organoids by fluorescence-based methods was reported by Jung et al., who transplanted fluorescently labeled HIOs under the kidney capsule and observed limited tissue penetration and high background [12]. Bergenheim et al. used confocal laser endomicroscopy with fluorescent dye labeling of HIOs, and found the signal was short lived and access to deeper structures was limited [13]. In contrast to these earlier reports, our BLI approach offers robust, longitudinal, and non-invasive tracking with minimal background, reflecting broader advancements in bioluminescence. This eliminates the need for potentially phototoxic illumination and enables deeper tissue signal detection, an important consideration for translational applications.

A persistent challenge in HIO xenotransplantation studies is confirming successful engraftment without waiting several weeks or sacrificing experimental animals [8], [25]. Here, the use of a red-shifted luciferase reporter overcame this limitation, allowing early detection and continuous monitoring without invasive procedures. Although our experiments were performed using the kidney capsule as the transplant site, the penetration of red-shifted bioluminescent light suggests that this approach could be readily adapted to other locations, such as the mesentery, where light attenuation through tissue might present more of a challenge to other modalities.

Repeated anesthesia and imaging can compromise murine well-being and induce some degree of physiological stress responses that may confound experimental outcomes [27], [28], [29], [30]. Stress-induced immunosuppression is known to affect tumor xenografts [27], [29], however similar effects have not been reported for non-neoplastic xenografts, such as human intestinal organoids (HIOs). Moreover, we are unaware of evidence that luciferin administration induces measurable physiological stress or perturbs the growth of organoids or other xenografts. Collectively, these observations support the use of bioluminescence imaging for longitudinal monitoring of HIOs, indicating that the procedure itself is unlikely to introduce significant experimental bias.

Serendipitously, luciferase expression became restricted to the epithelial compartment following differentiation into HIOs despite ubiquitous luciferase expression in the undifferentiated hESC state. We selected UbC because it is commonly used as a “constitutive” promoter; however, no promoter is uniformly active across all cell types and differentiation states [31]. Considering the semi-random nature of lentiviral integration, this lineage-specific restriction likely arises from integration into a genomic region subject to mesenchymal-specific heterochromatization. This interpretation is consistent with reports that UbC is broadly expressed across the human gastrointestinal tract yet shows higher expression in epithelial-rich tissues, including the stomach and small intestine [32], [33]. We acknowledge, however, that the absence of a parallel-differentiated, unmodified parental control line limits our ability to definitively exclude potential clonal artifacts or modification-related effects. This compartmentalization likely accounts for the relatively modest correlation between *in vivo* bioluminescence intensity (BLI) and total HIO explant mass. The BLI signal serves as a proxy for epithelial biomass, whereas gross measurements encompass mesenchymal and secreted components [34]. Future studies that directly quantify epithelial biomass and/or employ alternative promoters may strengthen the predictive relationship between BLI signal and tissue mass. Additionally, for applications requiring expression in a specific tissue compartment, targeted gene-editing tools (such as CRISPR-Cas9) could be employed to achieve more precise integration.

Epithelial-exclusive reporter expression offers several potential advantages in studying intestinal epithelial cell (IEC)-specific homeostasis in HIOs *in vitro* and *in vivo*; *e.g.*, screening compounds for IEC-specific responses within an *in vitro* HIO context; and tracking the engraftment of HIO-derived IECs into damaged gut epithelium *in vivo*. The compartmentalization of the bioluminescent reporter subsequent to differentiation, provides proof of principle that this approach is adaptable to study cell type–specific dynamics within xenotransplanted HIOs. By pairing lineage- or cell type–specific promoters with bioluminescent reporters, such a strategy could enable real-time, non-invasive monitoring of defined cellular populations *in vivo*, an approach that could be clinically relevant to many disease pathologies.

Of note, the capacity of the bioluminescent pluripotent hESC line to differentiate into HIOs and recapitulate the structural complexity of native intestine with both secretory and absorptive lineages, suggests that this engineering strategy preserves developmental potential. A current limitation, however, is the absence of functional validation regarding absorption, secretion, and enzymatic activity. Future studies must demonstrate that HIOs recapitulate these physiological processes to establish their utility for regenerative therapies.

In summary, we have developed a stable, pluripotent hESC line expressing a red-shifted luciferase reporter that supports efficient *in vitro* differentiation into HIOs and enables real-time, non-invasive *in vivo* monitoring of engraftment and growth. This approach addresses a critical gap in xenotransplantation research by providing a sensitive, reproducible, and adaptable tool for real-time assessment of human intestinal tissue implants. The ability to track transplanted tissues longitudinally and non-invasively will be invaluable for both mechanistic studies of organoid biology and the advancement of disease models and regenerative therapies.

## CRediT authorship contribution statement

**Chioma Moneme:** Writing – original draft, Investigation, Conceptualization. **Antonio Vinicios Alves da Silva:** Writing – review & editing, Writing – original draft, Visualization, Validation, Project administration, Methodology, Investigation, Formal analysis, Conceptualization. **Prisca C. Obidike:** Writing – original draft, Investigation, Formal analysis. **Bryan A. Hogg:** Investigation, Formal analysis. **Georgia B. Brousseau:** Investigation. **Casandra Robinson:** Project administration, Funding acquisition, Conceptualization. **Christian Roig-Laboy:** Investigation. **José Kleybson de Sousa:** Investigation. **Yuwen Zhang:** Investigation. **Lily S. Cheng:** Writing – original draft, Supervision, Methodology, Investigation, Formal analysis. **Sean R. Moore:** Writing – review & editing, Writing – original draft, Supervision, Project administration, Methodology, Investigation, Funding acquisition, Conceptualization.

## Declaration of Generative AI and AI-assisted technologies in the writing process

During the preparation of this work the author(s) used GPT 4o in order to improve language and readability. After using this tool/service, the author(s) reviewed and edited the content as needed and took full responsibility for the content of the publication.

## Funding/support

This Study was supported by 10.13039/100000865Bill and Melinda Gates Foundation (INV-039470).

CM and PO were supported by the NIH/NHLBI T32 training grant (T32HL007849).

LC was supported by NIH/NIDDK K08DK133673.

## Declaration of competing interest

All authors declare that they have no financial or personal relationships with other people or organisations that could inappropriately influence (bias) their work. No author has any competing interests to declare in relation to the submitted manuscript.

## Data Availability

All the data described in the manuscript are available from the corresponding author upon reasonable request.

## Appendix A Supplementary data

Supplementary Table 1 List of primary and secondary antibodies.
