# Supplementary material for: Non-invasive in vivo monitoring of transplanted human intestinal organoids using bioluminescence
Source: Surg Open Sci. 2026 May 15;32:8–16. doi: 10.1016/j.sopen.2026.05.002 (PMC13214524; doi:10.1016/j.sopen.2026.05.002)
Supplement: Supplementary Table 1 — List of primary and secondary antibodies. [file mmc2.docx]

**Supplementary Table 1. List of primary and secondary antibodies.**

| **Primary Antibody** | **Manufacturer** | **Catalog#** | **Dilution Used** |
| --- | --- | --- | --- |
| Luciferase | Promega | G7451 | 1:100 |
| Nanog | Abcam | AB21624 | 1:100 |
| Oct-3/4 | Santa-Cruz | 5280 | 1:100 |
| Sox2 | Santa-Cruz | sc-365823 | 1:250 |
| FoxA2 | R&D Systems | AF2400 | 1:100 |
| Sox17 | R&D Systems | MAB1924 | 1:100 |
| CDX-2 | Biogenex | MU392A-5UC | 1:50 |
| Muc-2 | Abcam | ab2272692 | 1:500 |
| Sucrase Isomaltase | Sigma-Millipore | HPA011897 | 1:200 |
| Chromogranin A | Roche Diagnostics | LK2H10 | 1 (Ready-to-Use) |
| Villin | Abcam | ab130751 | 1:100 |
| Lysozyme | Biorad | 0100-0523 | 1:100 |
|  |  |  |  |
| Secondary Antibody | Manufacturer | Catalog# | Dilution Used |
| Alexa 488 Donkey anti-Rabbit | Invitrogen | A21206 | 1:2000 |
| Alexa 488 Donkey anti-Goat | Invitrogen | A11055 | 1:2000 |
| Alexa 488 Donkey anti-Mouse | Abcam | ab150105 | 1:2000 |
| Alexa 546 Donkey anti-Rabbit | Invitrogen | A10040 | 1:2000 |
| Alexa 633 Goat anti Rabbit | Invitrogen | A-21070 | 1:1000 |
